# Could S-1-based non-platinum doublet chemotherapy be a new option as a second-line treatment for advanced non-small cell lung cancer patients? A multicenter retrospective study

**DOI:** 10.3389/fonc.2023.1089234

**Published:** 2023-03-16

**Authors:** Xiangling Wang, Ting Wang, Yunxia Chu, Jie Liu, Cuihua Yi, Xuejun Yu, Yonggang Wang, Tianying Zheng, Fangli Cao, Linli Qu, Bo Yu, Huayong Liu, Fei Ding, Shuang Wang, Xiangbo Wang, Jing Hao, Xiuwen Wang

**Affiliations:** ^1^ Department of Medical Oncology, Qilu Hospital of Shandong University, Jinan, China; ^2^ Department of Medical Oncology, Shandong Cancer Hospital, Jinan, China; ^3^ Department of Medical Oncology, Qingdao Branch of Qilu Hospital, Shandong University, Qingdao, China; ^4^ Department of Medical Oncology, Huantai People’s Hospital, Zibo, China; ^5^ Department of Medical Oncology, Linyi People’s Hospital, Linyi, China; ^6^ Department of Medical Oncology, The First People’s Hospital of Zibo, Zibo, China; ^7^ Department of Medical Oncology, Taian Central Hospital, Taian, China; ^8^ Department of Medical Oncology, Zhangqiu People’s Hospital, Jinan, China

**Keywords:** non-small cell lung cancer, second-line treatment, S-1, gemcitabine, docetaxel

## Abstract

**Background:**

For patients who have contraindications to or have failed checkpoint inhibitors, chemotherapy remains the standard second-line option to treat non-oncogene-addicted advanced non-small cell lung cancer (NSCLC). This study aimed to investigate the efficacy and safety of S-1-based non-platinum combination in advanced NSCLC patients who had failed platinum doublet chemotherapy.

**Methods:**

During January 2015 and May 2020, advanced NSCLC patients who received S-1 plus docetaxel or gemcitabine after the failure of platinum-based chemotherapy were consecutively retrieved from eight cancer centers. The primary endpoint was progression-free survival (PFS). The secondary endpoint was overall response rate (ORR), disease control rate (DCR), overall survival (OS), and safety. By using the method of matching-adjusted indirect comparison, the individual PFS and OS of included patients were adjusted by weight matching and then compared with those of the docetaxel arm in a balanced trial population (East Asia S-1 Trial in Lung Cancer).

**Results:**

A total of 87 patients met the inclusion criteria. The ORR was 22.89% (vs. 10% of historical control, *p* < 0.001) and the DCR was 80.72%. The median PFS and OS were 5.23 months (95% CI: 3.91–6.55 months) and 14.40 months (95% CI: 13.21–15.59 months), respectively. After matching with a balanced population in the docetaxel arm from the East Asia S-1 Trial in Lung Cancer, the weighted median PFS and OS were 7.90 months (vs. 2.89 months) and 19.37 months (vs. 12.52 months), respectively. Time to start of first subsequent therapy (TSFT) from first-line chemotherapy (TSFT > 9 months vs. TSFT ≤ 9 months) was an independent predictive factor of second-line PFS (8.7 months vs. 5.0 months, HR = 0.461, *p* = 0.049). The median OS in patients who achieved response was 23.5 months (95% CI: 11.8–31.6 months), which was significantly longer than those with stable disease (14.9 months, 95% CI: 12.9–19.4 months, *p* < 0.001) or progression (4.9 months, 95% CI: 3.2–9.5 months, *p* < 0.001). The most common adverse events were anemia (60.92%), nausea (55.17%), and leukocytopenia (33.33%).

**Conclusions:**

S-1-based non-platinum combination had promising efficacy and safety in advanced NSCLC patients who had failed platinum doublet chemotherapy, suggesting that it could be a favorable second-line treatment option.

## Introduction

Non-small cell lung cancer (NSCLC) is the most common type of primary lung cancer. Recent laboratory research highlighted current treatment strategies, such as radiotherapy, immunotherapy, and traditional therapy (1). In the clinic, especially for non-oncogene-addicted advanced NSCLC patients without contraindications to immune checkpoint inhibitors (ICIs), chemotherapy in combination with immunotherapy has become the preferred first-line treatment strategy. However, most patients will develop resistance to ICI over time. To date, several combination therapies are under development to delay or manage the acquired resistance to ICIs, including the blockade of immune coinhibitory signals, the activation of those with costimulatory functions, the modulation of the tumor microenvironment, and the targeting T-cell priming (2).

Thus far, conventional systemic chemotherapy alone or with antiangiogenic agents remains the mainstay treatment (3). To date, several options for second-line treatment are available, ranging from chemotherapy alone, in combination with an antiangiogenic agent, and immunotherapy. Docetaxel and pemetrexed are the most commonly used single agents. As a historical control, the response rate (RR) of docetaxel was approximately 10%, and the median progression-free survival (PFS) and overall survival (OS) were 2 to 4 months and 5.5 to 12.5 months, respectively (4–12). Since 2015, ICIs have become the preferred second-line option over docetaxel because of their significant improvement in OS, durable response, and better tolerability, with an ORR of 14% to 30%, a PFS of 2.3 to 4.0 months, and an OS of 9.2 to 13.8 months (7–10, 12, 13). However, the high cost of ICIs and low coverage of health insurance restrict ICI application in Chinese patients widely.

The roles of doublet chemotherapy in the second-line setting are still controversial. In one early meta-analysis (14), the combination arm showed a statistically significant improvement in ORR (15.1% vs. 7.3%) and PFS (14 weeks vs. 11.7 weeks), but no improvement difference in OS (37.3 weeks vs. 34.7 weeks) compared with single-agent therapy. However, most studies did not distinguish or categorize the patients by factors that might impact the potential benefit from the second-line combination chemotherapy. By contrast, platinum-based doublet chemotherapy was revealed to be the preferred second-line option over single-agent chemotherapy in clinical practice from two Chinese retrospective studies and conferred improved OS especially in patients with longer treatment-free interval (TFI) or time to progression from first-line chemotherapy (15, 16).

In East Asia, S-1 is another promising, well-tolerated, and cost-effective option in advanced NSCLC (17), which is a kind of oral compound anticancer drug composed of tegafur (FT), gimeracil (CDHP), and oteracil potassium (OXO). Furthermore, in comparison with pemetrexed, S-1 improved the synergistic therapeutic efficacy of anti-PD-1 antibodies by eliminating myeloid-derived suppressor cells and downregulating the expression of tumor-derived Bv8 and S100A8 (18). S-1 plus cisplatin or carboplatin demonstrated non-inferior OS as compared to paclitaxel or docetaxel plus platinum as first-line treatment in two randomized phase III trials (19, 20). Furthermore, S-1 was as effective as docetaxel in second-line therapy with less toxicity in the East Asia S-1 Trial in Lung Cancer (11). Several single-arm phase I/II trials evaluated the efficacy and safety of S-1 plus docetaxel or gemcitabine and had shown encouraging RR, OS, and non-overlapping toxicity profile (21–23). Data are still lacking about S-1 plus non-platinum doublet chemotherapy as a second-line option in Chinese advanced NSCLC patients. Therefore, we conducted a multicenter retrospective study to evaluate the efficacy and toxicity of S-1-based non-platinum combination chemotherapy in previously treated advanced NSCLC.

## Methods

### Patients

We retrieved data from the HIS (hospital information system) in the advanced NSCLC patients from January 2015 to May 2020 treated at eight institutions of Shandong Province in China, namely, Qilu Hospital of Shandong University, Qingdao branch of Qilu Hospital; Shandong University; Shandong Tumor Hospital; the First People’s Hospital of Zibo; Zhangqiu People’s Hospital; Linyi People’s Hospital; Taian Central People’s Hospital; and Huantai People’s Hospital.

Patients eligible for this study were required to meet the following criteria: (1) aged between 18 and 75 years; (2) ECOG performance status of 0 or 1; (3) histologically or cytologically confirmed NSCLC with stage IIIB or IV disease (AJCC 7th); (4) progression from first-line platinum-based doublet chemotherapy; (5) negative EGFR/ALK mutation or unknown before initiation of second-line chemotherapy; if EGFR/ALK mutation was positive, chemotherapy was introduced after TKI failure; (6) at least one measurable target lesion; (7) without symptomatic brain metastasis; and (8) received docetaxel plus S-1 or gemcitabine plus S-1 as second-line chemotherapy. The main exclusion criteria were as follows: (1) brain metastasis with symptoms; (2) patients with positive driver genes can be treated with other targeted therapies as a second-line treatment.

Approval for the retrospective review of these patients’ records was approved by the Medical Ethics Association of Qilu Hospital of Shandong University (2015040).

### Treatment

The chemotherapy regimen was docetaxel plus S-1 or gemcitabine plus S-1. The doses of drugs were within the following range: S-1: 40–60 mg, po, twice a day for 2 weeks and 1 week off; docetaxel: 60–75 mg/m², day 1 every 3 weeks; gemcitabine: 850–1,000 mg/m², day 1 and day 8 every 3 weeks. At least two cycles were required to evaluate the overall response rate (ORR). S-1 maintenance therapy was allowed for patients who achieved stable disease or response after combination chemotherapy.

### Study endpoints and assessments

The primary endpoint was PFS. The secondary endpoints were ORR, disease control rate (DCR), OS, and safety. Radiological response was assessed every 6 weeks in accordance with the Response Evaluation Criteria in Solid Tumors (RECIST 1.1). TSFT after first-line chemotherapy was calculated from the date of initiation of first-line chemotherapy to the initiation of second-line treatment. PFS was defined as the time period since the date of initiation of second-line therapy to the date of disease progression or death of any cause, whichever occurred first; OS was defined as the time period since the date of initiation of second-line therapy to the date of death of any cause or last follow-up. Adverse events were recorded according to Common Terminology Criteria for Adverse Events (version 4.0) of the National Cancer Institute. The subsequent treatment information after failure of second-line chemotherapy was attained by telephone interview or medical records. The last day of follow-up was 31 October 2020.

### Statistical analysis

The ORR of this study was compared with the ORR of docetaxel as a historical control by Fisher’s exact test, which was set as 10%, according to a summary of all phase III clinical studies of docetaxel as second-line treatment for patients with advanced NSCLC (**Table 1**). *p* < 0.025 in the unilateral test was considered statistically significant. Survival probabilities were calculated and compared in two groups using the Kaplan–Meier method and Log-rank test. Cox proportional hazards regression analysis was used to calculate the hazard ratio (HR) and 95% confidence interval (CI).

**Table 1:** Comparison of S-1-based doublet chemotherapy with docetaxel from phase III randomized controlled clinical trials of second-line treatment for advanced NSCLC.

Authors	Number	Treatment Arms	mPFS or TTP (months)	mOS (months)	ORR	1-year OS
Fossella et al. (4)	125 125 123	Docetaxel 100 mg/m² Docetaxel 75 mg/m² Vinorelbine or ifosfamide	8.4 weeks 8.5 weeks 7.9 weeks (*p* = 0.046)	5.5 5.7 5.6 (ns)	10.8% 6.7% 0.8%	21% 32% 19%
Shepherd et al. (5)	103 100	Docetaxel BSC	10.6 weeks 6.7 weeks (*p* ≤ 0.01)	7.0 4.6 (*p* = 0.47)	7.1% -	29% 19%
Hannal et al. (6)	288 283	Docetaxel 75 mg/m² Pemtrexed 500 mg/m²	2.9 2.9 (HR = 0.71, *p* = 0.759)	7.9 8.3 (HR = 0.99, *p* = 0.226)	8.8 9.1	29.7% 29.7%
Borghaei et al. (7)	292 290	Nivolumab3 mg/kg Docetaxel 75 mg/m²	2.3 4.2	12.2 9.4 (HR = 0.73, *p* = 0.002)	19% 12% (*p* = 0.02)	51% 39%
Brahmer et al. (8)	135 137	Nivolumab 3 mg/kg Docetaxel 75 mg/m²	3.5 2.8	9.2 6.0	20% 9% (*p* = 0.008)	42% 24%
Herbst et al. (9)	344 346 343	Pembrolizumab 2 mg/kg Pembrolizumab 10 mg/kg Docetaxel 75 mg/m²	3.9 (HR = 0.88, *p* = 0.07) 4.0 (HR = 0.79, *p* < 0.004) 4.0	10.4 (HR = 0.71, *p* = 0.0008) 12.7 (HR = 0.61, *p* < 0.0001) 8.5	30% 29% 8%	43.2% 52.3% 34.6%
Rittmeyer et al. (10)	425 425	Atezolizumab 1,200 mg Docetaxel 75 mg/m²	2.8 (HR = 0.95) 4.0	13.8 9.6 (HR = 0.73, *p* = 0.0003)	14% 13%	
Nokihara et al. (11)	577 577	Docetaxel 60 or 75 mg/m² S-1 80–120 mg/day	2.89 2.86 (HR = 1.033, *p* = 0.608)	12.52 12.75 (HR = 0.945, *p* = 0.3818)	9.9% 8.3%	
Wu et al. (12)	338 166	Nivolumab 3 mg/kg Docetaxel 75 mg/m²	2.8 2.8 (HR = 0.77, *p* = 0.0147)	12.0 9.6 (HR = 0.68, *p* = 0.0006)	17% 4%	50% 39%
Takiguchi et al. (21)	34	Gemcitabine 1,000 mg/m² d8,15 + S-1 60 mg/m²/day, d1–14	6.6	19.9	23.5%	58.8%
Yanagihara et al. (22)	38	Docetaxel 40 mg/m² d1+ S-1 40–60 mg, bid, d1–14	4.4	16.1	18.4%	60.0%
Atagi et al. (23)	30	Docetaxel 40 mg/m² d1+ S-1 80 mg/m²/day, d1–14	3.9	11.8	24.1%	41.8%
Segawa et al. (24)	31 29	Docetaxel 40 mg/m² d1+ S-1 80 mg/m²/day, d1–14 Docetaxel 60 mg/m² d1	3.4 3.7	8.7 22.9 (HR = 0.42, *p* = 0.02)	16.1% 20.7%	33.5% 73.8%
This study	87	Docetaxel 60–75 mg/m² or Gemcitabine 850–1,000 mg/m², d1, 8 + S-1 40–60 mg, bid, d1–14	5.23	14.40	22.89%	62.6%

TTP, time to progress.

The PFS and OS of this study were compared with those of docetaxel using matching-adjusted indirect comparison (MAIC). After a systematic literature review, patients across our study and the East Asia S-1 Trial in Lung Cancer were matched on a range of potential effect modifiers, including age, sex, smoking status, TNM stage, histology, postoperative recurrence, ECOG performance status, and EGFR mutation status (11). Data from patients receiving S-1 were re-weighted to match the baseline characteristics of patients included in the East Asia S-1 Trial receiving docetaxel. The individual weights were estimated using a logistic model as described by Signorovitch et al. (25). OS and PFS analyses were conducted using aggregate data. All statistical analyses were carried out by R 3.5.0 and SAS 9.4 software, and a two-sided test was performed (*α* = 0.05).

## Results

### Patient characteristics

**Figure 1:**
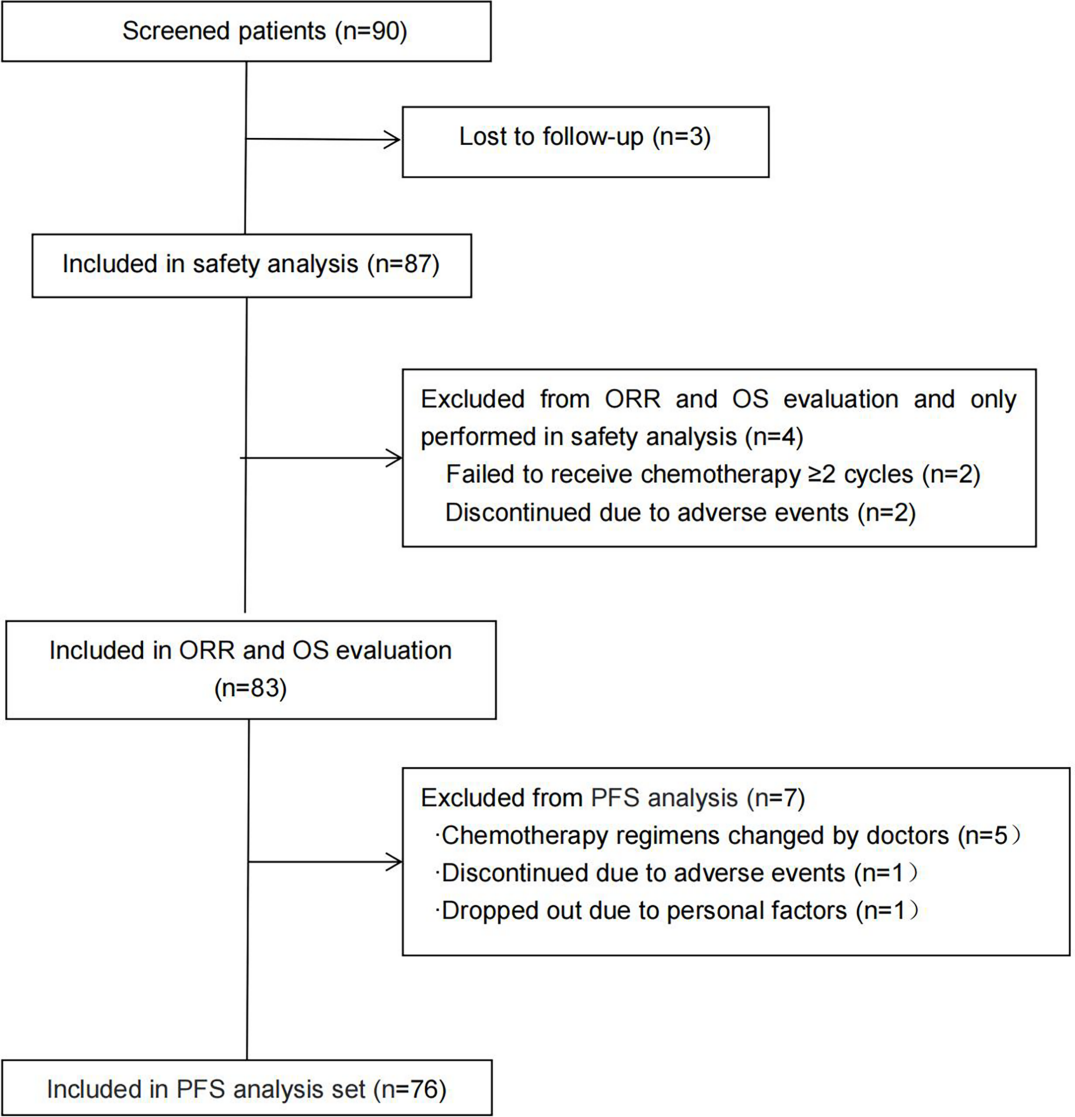
Consort diagram: patient flow for safety, ORR, PFS, and OS.

A total of 87 patients were eligible for safety analysis; 34 patients (39.08%) received gemcitabine plus S-1, while 53 cases (60.92%) were given docetaxel combined with S-1. The median follow-up time was 14.13 months, and the median treatment course was 4 cycles (range, 1–12). A total of 83 patients were eligible for ORR and OS analysis and 76 patients were eligible for PFS analysis (**Figure 1**). The clinicopathological characteristics of 83 cases are shown in **Table 2**. Two-thirds of patients were men and the median age was 60 years. Nearly one-fourth of patients were stage IIIB and one-third were squamous carcinomas. Most patients (78%) had ECOG PS 1. Less than 10% of the patients had asymptomatic brain metastasis. EGFR and ALK mutations were seen in 5 and 3 out of 52 patients, respectively, who had gene mutation detection before enrollment. The time to first subsequent therapy (TFST) of first-line chemotherapy in 83 patients was 4.6 months (2.1–8.7 months) and 17 patients (20.48%) had more than 9 months of mPFS (**Table 2**). Most patients (84.2%) were treated at the Qilu Hospital of Shandong University.

**Table 2:** Univariate and multivariate Cox regression analysis of PFS in 76 advanced NSCLC patients receiving S-1-based non-platinum doublet chemotherapy as a second-line treatment.

Characteristics	*N* (%)	mPFS (months) (*N* = 76)	*p*	Multivariate analysis		
				HR	95% CI	*p*
Age (years)						
<60	35 (46.05%)	5.2 (2.6–7.9)	0.600	Ref		
≥60	41 (53.95%)	5.2 (4.6–5.8)		1.606	0.884–2.920	0.120
Gender						
Female	26 (34.21%)	4.5 (3.0–6.0)	0.578	Ref		
Male	50 (65.79%)	5.4 (3.6–7.3)		1.631	0.574–4.635	0.359
Smoking status						
Never	36 (47.37%)	5.2 (4.1–6.3)	0.824	Ref		
Current/Ever	40 (52.63%)	6.6 (4.3–9.0)		0.609	0.224–1.656	0.331
TNM Stage						
Stage IIIB	15 (19.74%)	4.8 (3.1–6.5)	0.710	Ref		
Stage IV	61 (80.26%)	5.2 (3.8–6.7)		0.848	0.343–2.098	0.721
Histology						
Non squamous	50 (65.79%)	5.2 (3.3–7.1)	0.365	Ref		
Squamous	26 (34.21%)	5.4 (4.7–6.2)		0.415	0.148–1.161	0.094
Postoperative recurrence						
No	59 (77.63%)	5.2 (3.7–6.7)	0.820	Ref		
Yes	17 (22.37%)	5.4 (2.1–8.7)		0.972	0.506–1.868	0.932
Brain metastases						
No	69 (91.79%)	5.4 (3.7–7.2)	0.857	Ref		
Yes	7 (9.21%)	3.9 (0.0–8.3)		0.762	0.291–1.995	0.580
ECOG performance status						
0	16 (21.05%)	5.0 (1.9–8.1)	0.593	Ref		
1	60 (78.95%)	5.2 (3.6–6.9)		1.001	0.500–2.002	0.998
Mutation status						
Positive	7 (9.21%)	8.9 (4.2–11.9)	0.584	Ref		
Negative	40 (52.63%)	4.5 (3.2–5.7)		1.281	0.645–2.545	0.479
Unknown	29 (38.16%)	5.4 (4.8–6.1)				
Hospital						
Qilu Hospital of Shandong University	64 (84.21%)	5.2 (4.2–6.2)	0.659	Ref		
Other	12 (15.79%)	7.9 (3.3–12.5)		0.835	0.375–1.860	0.659
PFS of first-line treatment						
≤9 months	59 (77.63%)	4.9 (4.2–5.7)	0.042	Ref		
>9 months	17 (22.37%)	8.7 (7.3–10.0)		0.437	0.211–0.905	0.026
Second-line chemotherapy regimens						
Docetaxel + S-1	46 (60.53%)	5.4 (3.6–7.3)	0.146	Ref		
Gemcitabine + S-1	30 (39.47%)	4.4 (3.6–5.3)		1.501	0.829–2.717	0.180

### Efficacy

A total of 19 patients (22.89%) were evaluated as partial remission (PR) among 83 patients, much higher than the 10% RR with docetaxel in the historical control (*p* < 0.001). A total of 48 patients (57.83%) achieved stable disease and 16 patients (19.28%) progressed. The DCR was 80.72%. Of note, the ORR was 52.94% (9/17) in patients with a TFST of > 9 months, whereas in those with a TFST of ≤ 9 months, the ORR was 16.95% (10/59), *p* = 0.007.

**Figure 2:**
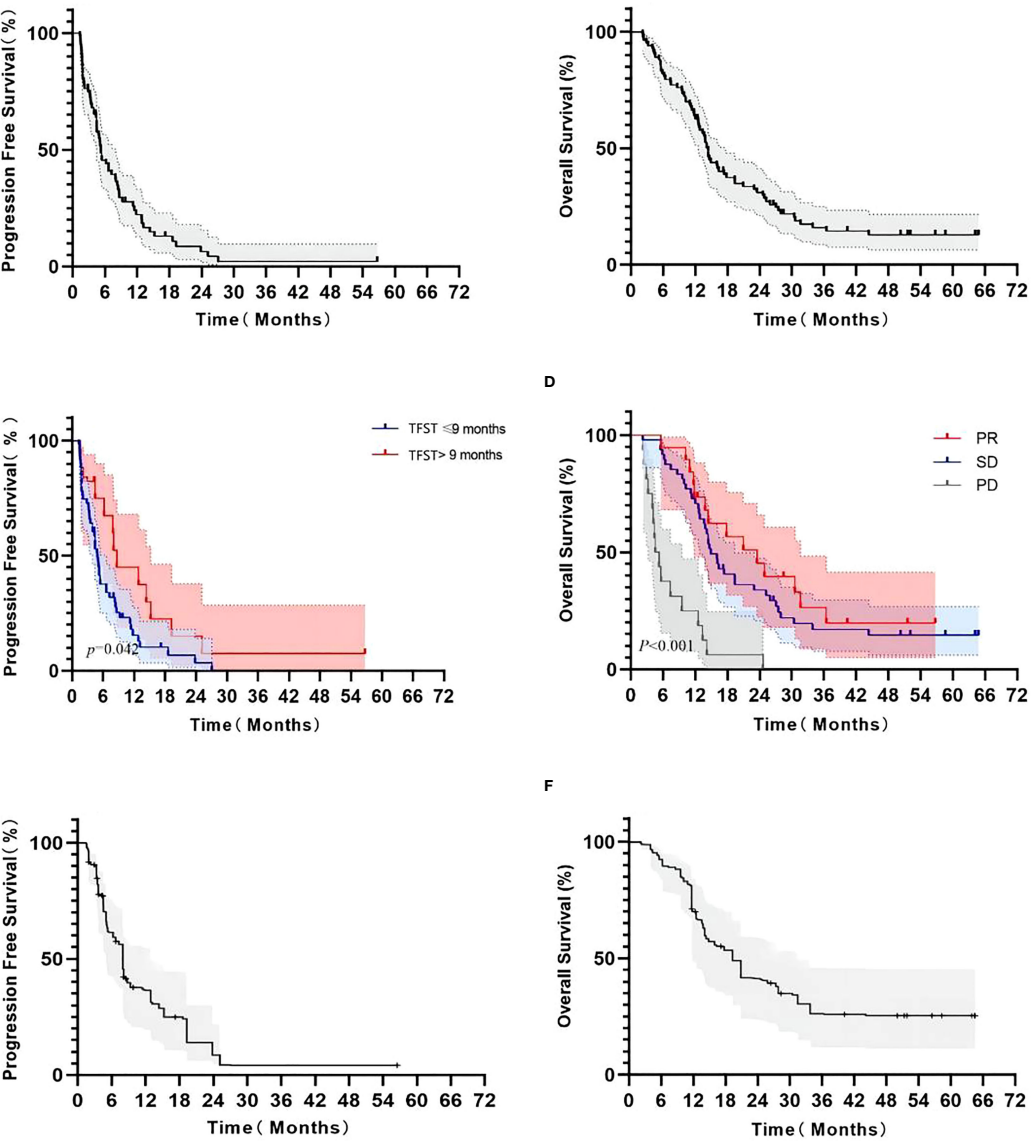
Kaplan–Meier survival curve in advanced NSCLC patients receiving second-line S-1-based doublet chemotherapy. Progression-free survival **(A)**; overall survival **(B)**; PFS according to the TFST from first-line chemotherapy (>9 months and ≤9 months, **(C)**; OS according to response from second-line chemotherapy (PR, SD, and PD, **(D)**; adjusted PFS by weight matching **(E)**; adjusted OS by weight matching **(F)**.

Of 76 patients, 74 (97.4%) had disease progression. Median PFS was 5.23 months (95% CI: 3.91–6.55 months). Six-month PFS rate was 45.5% (95% CI: 33.6%–56.7%) and 1-year PFS rate was 22.2% (95% CI: 12.8%–33.2%), as shown in **Figure 2**. Multivariate Cox analysis showed that a TFST of > 9 months of first-line chemotherapy was an independent favorable predictive factor of second-line PFS (8.7 months vs. 4.9 months, HR = 0.437, *p* = 0.026) (**Table 2**).

By the end of follow-up, 69 patients (83.1%) died from cancer. The median OS was 14.40 months (95% CI: 13.21–15.59 months). Six-month OS rate was 81.9% (95% CI: 71.8%–88.7%) and 1-year OS rate was 62.6% (95% CI: 51.3%–72.0%), as shown in **Figure 2**. The median OS in patients who achieved response was 23.5 months (95% CI: 11.8–31.6 months, *p* < 0.001), which was significantly longer than those with stable disease (14.9 months, 95% CI: 12.9–19.4 months) or progression (4.9 months, 95% CI: 3.2–9.5 months) (**Table 3**).

**Table 3:** Univariate and multivariate Cox regression analysis of OS in 83 advanced NSCLC receiving S-1 based doublet as second line treatment.

Characteristics	N (%)	mOS (month)(N = 83)	*P*	Multivariate analysis		
				HR	95% CI	*p*
Age (years)						
**< 60**	38 (45.78%)	13.4 (9.6-17.1)	0.574	Ref		
≥ 60	45 (54.21%)	14.9 (11.2-18.6)		0.820	0.452-1.485	0.512
Gender						
Female	28 (33.73%)	13.8 (9.9-17.7)	0.978	Ref		
Male	55 (66.27%)	14.4 (13.3-15.5)		0.777	0.376-1.609	0.498
Smoking status						
Never	38 (45.79%)	13.8 (11.0-16.6)	0.927	Ref		
Current/Ever	45 (54.21%)	14.4 (11.7-17.2)		1.325	0.567-3.094	0.516
TNM Stage						
Stage IIIB	20 (24.10%)	14.1 (11.9-16.4)	0.851	Ref		
Stage IV	63 (75.90%)	14.4 (13.1-15.7)		1.543	0.698-3.411	0.283
Histology						
Non squamous	54 (65.06%)	14.1 (11.4-16.9)	0.318	Ref		
Squamous	29 (34.94%)	14.4 (12.4-16.4)		2.218	1.015-4.846	0.046
Postoperative recurrence						
NO	65 (78.31%)	14.4 (13.2-15.6)	0.560	Ref		
Yes	18 (21.69%)	11.2 (1.5-20.8)		1.538	0.765-3.091	0.227
Brain metastases						
NO	76 (91.57%)	14.4 (12.3-16.5)	0.317	Ref		
Yes	7 (8.43%)	7.4 (2.9-11.8)		1.075	0.408-2.829	0.884
ECOG performance status						
0	18 (21.69%)	16.0 (12.7-33.9)	0.261	Ref		
1	65 (78.31%)	14.1 (11.7-16.4)		1.596	0.779-3.269	0.201
Mutation status						
positive	8 (9.64%)	14.1 (11.0-24.9)	0.721	Ref		
negative	44 (53.01%)	15.9 (9.5-31.6)		0.874	0.496-1.539	0.641
unknown	31 (37.35%)	13.7 (8.7-17.8)				
Hospital						
Qilu Hospital of Shandong University	71 (85.54%)	14.1 (12.4-15.9)	0.105	Ref		
other	12 (14.46%)	17.4 (7.9-26.9)		0.546	0.244-1.223	0.141
PFS of First-line treatment						
≤ 9 months	65 (78.31%)	14.4 (13.4-15.4)	0.047	Ref		
> 9 months	18 (21.69%)	14.1 (4.4-23.9)		0.612	0.279-1.341	0.219
Response to second-line chemotherapy						
PR	19 (22.89%)	23.5 (13.9-33.1)	<0.001	Ref		
SD	48 (57.83%)	14.9 (12.7-17.1)		2.716	1.705-4.327	<0.001
PD	16 (19.28%)	4.9 (2.8-6.3)				
Second line chemotherapy regimens						
Docetaxel+S-1	49 (59.04%)	13.8 (12.0-15.6)	0.839	Ref		
Gemcitabine+S-1	34 (40.96%)	16.0 (10.0-22.0)		0.706	0.405-1.231	0.220

When adjusted by weight matching with a weighted balanced population from the East Asia S-1 Trial in Lung Cancer (11), 37.9 and 39.4 of the patients were used for comparison for PFS and OS, respectively (**Table 4**). The weighted mPFS and mOS was 7.90 months (**Figure 2A**) and 19.37 months (**Figures 2E, F**), respectively, with S-1 combined with non-platinum-based regimen. A 95% CI of mPFS and mOS was not available owing to the small number of enrolled patients after weighting.

**Table 4:** Indirect adjustment of PFS and OS by weight matching.

Characteristics	Docetaxel (*N* = 570)	S-1 plus non-platinum before adjusting (*N* = 76)	S-1 plus non-platinum after adjusting (*N* = 37.9)	S-1 plus non-platinum before adjusting (*N* = 83)	S-1 plus non-platinum before adjusting (*N* = 39.4)
Age (years), median (range)	62 (28, 82)	60 (53, 67)	62 (56, 68)	60 (53, 67)	60 (53, 67)
Gender					
Male	381 (66.8%)	50 (65.8%)	25.3 (66.8%)	55 (66.3%)	55 (66.3%)
Female	189 (33.2%)	26 (34.2%)	12.6 (33.2%)	28 (33.7%)	28 (33.7%)
Smoking status					
Current/Ever	383 (67.2%)	40 (52.6%)	25.5 (67.2%)	45 (54.2%)	45 (54.2%)
Never	187 (32.8%)	36 (47.4%)	12.4 (32.8%)	38 (45.8%)	38 (45.8%)
TNM Stage					
Stage IIIB	35 (6.1%)	15 (19.7%)	2.3 (6.1%)	20 (24.1%)	20 (24.1%)
Stage IV	535 (93.9%)	61 (80.3%)	35.6 (93.9%)	63 (75.9%)	63 (75.9%)
Histology					
Squamous	97 (17.0%)	26 (34.2%)	6.4 (17.0%)	29 (34.9%)	29 (34.9%)
Non-squamous	473 (83.0%)	50 (65.8%)	31.5 (83.0%)	54 (65.1%)	54 (65.1%)
Postoperative recurrence					
Yes	114 (20.0%)	17 (22.4%)	7.6 (20.0%)	18 (21.7%)	18 (21.7%)
No	456 (80.0%)	59 (77.6%)	30.3 (80.0%)	65 (78.3%)	65 (78.3%)
ECOG performance status					
0	207 (36.3%)	16 (21.1%)	13.8 (36.3%)	18 (21.7%)	18 (21.7%)
1 or 2	363 (63.7%)	60 (78.9%)	24.1 (63.7%)	65 (78.3%)	65 (78.3%)
EGFR/ALK mutation					
Positive	130 (22.8%)	4 (5.3%)	8.6 (22.8%)	4 (4.8%)	9.0 (22.8%)
Negative/unknown	440 (77.2%)	72 (94.7%)	29.3 (77.2%)	79 (95.2%)	30.4 (77.2%)
mPFS (months, 95% CI) mOS (months, 95% CI)	2.86 (2.73–3.12) 12.75 (11.53–14.00)	5.23 (4.40–7.90)	7.90 (NA)	14.40 (12.53–17.40)	19.37 (NA)

At the date of last follow-up, two patients were still free from progression. Fourteen patients only received best supportive care after failure of chemotherapy. A total of 65 patients (82.28%) received subsequent systemic treatment (**Table 5**), in which chemotherapy alone accounted for 31.64% of the cases and antiangiogenic tyrosine kinase inhibitor accounted for 27.85%. Five patients each received later-line anti-PD-1/PD-L1 therapy and further targeted therapy.

**Table 5:** Subsequent treatments after second-line therapy.

Subsequent treatments (*n* = 79)	*n* (%)
Chemotherapy alone	25 (31.64)
Antiangiogenic TKI alone Anlotinib Apatinib	16 (20.25) 8 (10.13) 8 (10.13)
Chemotherapy plus antiangiogenic therapy Chemotherapy + bevacizumab Chemotherapy + anlotinib Chemotherapy + apatinib	6 (7.60) 4 (5.06) 1 (1.27) 1 (1.27)
Targeted therapy EGFR-TKI ALK-TKI	5 (6.33) 3 (3.80) 2 (2.53)
Radiotherapy	8 (10.13)
Immunotherapy Anti-PD-1/PD-L1 therapy Anti-PD-1 therapy plus antiangiogenic TKI	5 (6.33) 1 (1.27) 4 (5.06)
Best support care	14 (17.72)

### Safety

As shown in **Table 6**, a total of 87 patients were evaluated for safety; adverse events of any grade in our study were 88.51%. Grade 3–4 adverse events occurred in 11 of 53 patients (20.75%) in the S-1 plus docetaxel subgroup and in 7 of 34 patients (20.59%) in the S-1 plus gemcitabine subgroup. Ten patients (11.49%) experienced dose reduction and four patients (4.60%) interrupted the treatment.

**Table 6:** Adverse events of S-1-based doublet chemotherapy.

Group	S-1 + non-platinum (*N* = 87)		S-1 + gemcitabine (*N* = 34)		S-1 + docetaxel (*N* = 53)	
	Any grade (*n*, %)	Grades 3–4 (*n*, %)	Any grade (*n*, %)	Grades 3–4 (*n*, %)	Any grade (*n*, %)	Grades 3–4 (*n*, %)
Adverse events	77 (88.51%)	18 (20.69%)	30 (88.24%)	7 (20.59%)	47 (88.68%)	11 (20.75%)
Anemia	53 (60.92%)	1 (1.15%)	24 (70.59%)	0 (0)	29 (54.72%)	1 (1.89%)
Neutropenia	25 (28.74%)	13 (14.94%)	10 (29.41%)	5 (14.71%)	15 (28.30%)	8 (15.09%)
Thrombocytopenia	16 (18.39%)	3 (3.45%)	10 (29.41%)	2 (5.88%)	6 (11.32%)	1 (1.89%)
Leukocytopenia	29 (33.33%)	7 (8.05%)	13 (38.24%)	0 (0)	16 (30.19%)	7 (13.21%)
Alanine aminotransferase increased	6 (6.90%)	1 (1.15%)	1 (2.94%)	0 (0)	5 (9.43%)	1 (1.89%)
Aspartate aminotransferase increased	12 (13.79%)	1 (1.15%)	3 (8.82%)	1 (2.94%)	9 (16.98%)	0 (0)
Blood bilirubin increased	13 (14.94%)	0 (0)	5 (14.71%)	0 (0)	8 (15.09%)	0 (0)
Creatinine increased	0 (0)	0 (0)	0 (0)	0 (0)	0 (0)	0 (0)
Nausea	48 (55.17%)	1 (1.15%)	20 (58.82%)	0 (0)	28 (52.83%)	1 (1.89%)
Vomiting	11 (12.64%)	0 (0)	5 (14.71%)	0 (0)	6 (11.32%)	0 (0)
Diarrhea	3 (3.45%)	1 (1.15%)	1 (2.94%)	0 (0)	2 (3.77%)	1 (1.89%)
Alopecia	11 (12.64%)	0 (0)	6 (17.65%)	0 (0)	5 (9.43%)	0 (0)
Rash	6 (6.90%)	0 (0)	3 (8.82%)	0 (0)	3 (5.66%)	0 (0)
Skin hyperpigmentation	8 (9.20%)	0 (0)	2 (5.88%)	0 (0)	6 (11.32%)	0 (0)

There was no treatment-related death. The most common adverse events were anemia (60.92%), nausea (55.17%), and leukocytopenia (33.33%), whether in the docetaxel or the gemcitabine plus S-1 subgroup. The most common grade 3/4 adverse event was neutropenia (14.94%).

## Discussion

To our knowledge, this study was the first to explore the efficacy and safety of S-1-based non-platinum combination chemotherapy as a second-line treatment in non-oncogene-addicted advanced Chinese NSCLC patients. There were two major findings.

The first finding was that S-1-based non-platinum combination chemotherapy could be a promising second-line option. When compared with docetaxel as a historical control, as shown in **Table 1**, this multicenter study provided encouraging benefit in response (22.89%), PFS (5.23 months), OS (14.40 months), and good tolerability. After adjustment by weight matching, there was still a dramatic improvement in PFS (7.90 months) and OS (19.37 months). The results of our study were consistent with most of the other Japanese cohort studies (21–23). The excellent ORR and PFS might be attributed to the combination of S-1 and docetaxel or gemcitabine and also S-1 maintenance. Furthermore, the median OS in patients who achieved response was significantly longer than those with stable disease or progression, which suggested the durable survival benefit from S-1-based doublet non-platinum chemotherapy once achieving response.

One exception was OLCSG trial 0503 (24), in which the single-agent docetaxel had a similar ORR and PFS, but an extraordinarily prolonged survival compared with docetaxel plus S-1 (22.9 months vs. 8.7 months). The much higher percentages of patients receiving poststudy EGFR TKI in the single docetaxel arm (42.9% vs. 16.7%) might contribute to this discrepancy. Poststudy treatment was very prevalent (81.82%) in our study, and nearly 30% of the patients received antiangiogenic treatment with bevacizumab, apatinib, or anlotinib. All of the above post-study drugs were considered to prolong the OS. In a recently published study (26), anlotinib plus S-1 as third- or later-line treatment showed very promising antitumor activity, with an ORR of 37.9%, a PFS of 5.8 months, and an OS of 16.7 months, supporting the use of S-1 and anlotinib either concomitantly or subsequently in the future. Also, in another prospective study (27), bevacizumab and S-1 combination chemotherapy showed high activity with an ORR of 28.3%, a PFS of 4.3 months, and an OS of 15.0 months, and tolerable toxicities.

The second finding was that TFST could predict the PFS benefit from S-1 doublet chemotherapy. The patients with a TFST of > 9 months had a much longer PFS (8.7 months) than those with a TFST of ≤ 9 months (5.0 months). In other words, TFST from first-line chemotherapy could distinguish the patients who might benefit the most from second-line therapy, which was confirmed in two previous Chinese retrospective studies (15, 16). Both a TFST of > 12 months and a TFI of > 6 months from first-line chemotherapy were independent predictive factors of favorable PFS and OS in advanced NSCLC patients who received platinum-based doublet chemotherapy in the second-line setting. With additional new drugs introduced into the first-line treatment in advanced NSCLC, especially CPIs (checkpoint inhibitors) and antiangiogenic agents, whether TFST from these new first-line combinations could predict the benefit of the subsequent treatment remains to be elucidated in the future.

Regarding safety, both hematological and non-hematological toxicity in our study were tolerable and could be well controlled through treatment interruption and/or symptomatic treatment and dose reduction. There were no treatment-related deaths. Common adverse events of S-1 with docetaxel or gemcitabine were anemia, nausea, and leukocytopenia. The most common grade 3/4 adverse event was neutropenia (14.94%). All the above toxicities were higher than those of docetaxel as a historical control (3–10), but consistent with the data of previous S-1 studies (11, 21–23).

New evidence showing that immunotherapy plus chemotherapy could substantially improve ORR, PFS, and OS when compared with immunotherapy (4) or chemotherapy (28, 29) alone has emerged. S-1 had been proven to have a synergistic effect with nivolumab in gastric cancer (30); thus, it can be expected that S-1 would be introduced to clinical trials of immunotherapy in advanced NSCLC in the future.

The present study had some limitations. First, our study is limited by its retrospective nature and patients’ heterogeneity. Selection bias could not be ignored, since patients who could not afford the cost of ICIs or refused the gene mutation analysis had more chances of receiving cytotoxic drugs as a second-line treatment. Second, during the study period, no patient received ICIs in the first-line setting; whether the efficacy of S-1-based doublet chemotherapy as second-line option could be generalized to those who failed immunotherapy needs to be explored. Actually, there may be no need to worry about it since subsequent S-1 or docetaxel after nivolumab was proved to be more effective than regimens without ICI pretreatment (31). Moreover, the high percentage of unknown driver mutation in our study precluded patients’ availability to targeted therapy. Indeed, in the NJLCG0804 trial (32), among NSCLC patients whose treatment with epidermal growth factor receptor tyrosine kinase inhibitors and platinum-based chemotherapy failed, S-1 and irinotecan combination therapy demonstrated high effectiveness; the ORR reached 52.0%, and PFS and OS were 5.0 and 17.1 months, respectively. Third, although we did not find a significant difference in survival benefit among patients from different centers, it should be noted that most patients in this study were from the sponsor’s cancer center, while platinum-based doublet chemotherapy as second-line option seemed to be more popular in other institutions from the same province (15).

In conclusion, S-1-based non-platinum combination had promising efficacy and manageable toxicity in advanced NSCLC patients who had failed platinum doublet chemotherapy, indicating a favorable second-line option.

## Data availability statement

The original contributions presented in the study are included in the article/supplementary material. Further inquiries can be directed to the corresponding authors.

## Ethics statement

The studies involving human participants were reviewed and approved by the Medical Ethics Association of Qilu Hospital of Shandong University (2015040). Written informed consent for participation was not required for this study in accordance with the national legislation and the institutional requirements.

## Author contributions

Conception and design: JH and XWW; Administrative support: XWW; Provision of study materials or patients: TW, XLW, YC, JL, CY, XY, YW, TZ, FC, LQ, BY, HL, FD, SW, XBW, JH, and XWW; Collection and assembly of data: XLW, TW, and JH; Data analysis and interpretation: XLW, TW, and JH; Manuscript writing: All authors; All authors contributed to the article and approved the submitted version.
